# Society Meetings

**Published:** 1914-02

**Authors:** 


### SOCIETY MEETINGS.

*Brief reports and announcements of meetings of societies of Western New York and those of general scope are requested from Secretaries. Copy should be on hand the fifteenth of each month. Full transactions will be published at cost of composition.*

Buffalo Academy of Medicine: Pathology, December 23, 1913, "Beri-Beri," Maj. F. H. Wadhams, M. D., of Fort Porter, Buffalo; Surgery, January 6, "Good Lighting and How to Obtain It," M. Luckiesh of the Physical Laboratory, National Electric Lamp Association, Cleveland; Stated Meeting under auspices of Section of Medicine, January 13, Lantern Slide Illustrations of Aphasia, Dr. August Hoch, Director of Pathologic Institute of State Hospitals, New York; Obstetrics and Gynaecology, January 20, "Syphilis as a Complication of Pregnancy," Wm. T. Getman, M. D., discussion by Nelson W. Wilson, M. D.; "Vaginal Caesarian Section in Eclampsia," Irving W. Potter, M. D., discussion by Matthew D. Mann, M. D, all of Buffalo except as specified.

Elmira Academy of Medicine, January 7, 1914, Prof. James M. Caird of Troy spoke on "Water Filtration, Ancient and Modern," illustrated by 100 lantern slides.

Rochester Academy of Medicine, Nominations: For President, William B. Jones, M. D., Charles R. Barber, M. D., Charles Dean Young, M. D.; For Four Vice-Presidents, Willard H. Veeder, M. D., George G. Carroll, M. D., James K. Quigley, M. D., John M. Swan, M. D.; For Treasurer, Wesley T. Mulligan, M. D.; For Three Trustees, John F. W. Whitbeck, M. D., Nathan W. Soble, M. D., Montgomery E. Leary, M. D.; For Two Councillors, William V. Ewers, M. D., Myron B. Palmer, M. D., J. W. Magill, M. D., For Two Members of Library Committee, John M. Swan, M. D., Joseph Roby, M. D., Charles W. Hennington, M. D. Address of Retiring President, Dr. Charles E. Darrow, President; Dr. Edward L. Hanes, Secretary.

---

At its first quarterly meeting, January 6, 1914, at Salamanca, N. Y., the Cattaraugus County Medical Society has elected the following officers: President, Dr. H. W. Johnson, Gowanda; Vice-President, Dr. R. B. Morris, Olean; Secretary-Treasurer, Dr. H. H. Ashley, Machias; Delegate to State Society, Dr. H. W. Johnson, Gowanda; Alternate, Dr. E. Torrey, Olean; Delegate 8th District Branch, Dr. F. C. Beals, Salamanca; Alternate, Dr. M. C. Hawley, Randolph; Censors, Dr. S. S. Bedient, Little Valley; Dr. A. D. Lake, Gowanda; Dr. E. Torrey, Olean; Dr. M. E. Fisher, Delevan; Dr. F. C. Beals, Salamanca.

---

The Buffalo Medical and Surgical League had its January meeting at the Lutheran Church Home. Dr. Alfred Noehren, the attending physician, gave a clinic on senile diseases.

---

Ontario County Medical Society met in Canandaigua January 13th. The President's Address: "Tuberculosis of the Peritoneum" by Dr. S. R. Wheeler, East Bloomfield. "Differential Temperature in Otology," Dr. George F. Cott, Buffalo. "Influensal Pneumonia," Dr. H. J. Q. Howe, Phelps.

---

Canandaigua Society of Physicians and Surgeons met on January 8th, at the Canandaigua Hotel. The President's Address: "Present Status of Vaccine Therapy" by Dr. A. W. Armstrong. Officers elected: Dr. H. C. Buell, President; Dr. A. L. Beahan, Vice-President; Dr. H. C. Burgess, Secretary-Treasurer. Dr. A. M. Mead of Victor was the host.

